# Comprehensive Transcriptomic Profiling of Diverse Brain Tumor Types Uncovers Complex Structures of the Brain Tumor Microenvironment

**DOI:** 10.3390/biomedicines12030506

**Published:** 2024-02-23

**Authors:** Jiin Choi, Hee Jin Cho

**Affiliations:** 1 Department of Biomedical Convergence Science and Technology, Kyungpook National University, Daegu 41566, Republic of Korea; djm04018@knu.ac.kr; 2 Cell and Matrix Research Institute, Kyungpook National University, Daegu 41944, Republic of Korea

**Keywords:** brain tumor, tumor microenvironment, transcriptome analysis, single-cell RNA sequencing

## Abstract

Various types of brain tumors occur in both children and adults. These tumors manifest with different characteristics such as malignancy, cellular lineage, location of origin, and genomic profile. Recently, immunotherapy, which manipulates immune cells in the tumor microenvironment (TME) to kill tumor cells, has attracted attention as a treatment strategy for tumors. Here, we analyzed the transcriptomic architecture of the brain tumor microenvironment to provide potential guidelines to overcome the therapeutic vulnerabilities to brain tumors. We decomposed the cellular populations of six brain tumor types (meningioma, pilocytic astrocytoma, ependymoma, medulloblastoma, glioblastoma, and lower-grade glioma) using publicly available microarray data and single-cell RNA sequencing (scRNA-seq) data. Interestingly, transcriptome-based immune cell profiling revealed that infiltrating immune cell types in the brain TME, particularly M2 macrophages, CD8+ T cells, and CD4+ T cells, could be distinguished by tumor type, malignancy, and location. scRNA-seq revealed differences in the proportions of dendritic and mural cells. Unsupervised clustering using immune-related genes divided all samples into two distinct clusters with different characteristics. In addition, immune subpopulations showed disparate reactions after anti-PD-1 therapy for glioblastoma. Our results unveiled the distinct TME across brain tumor types and provided a transcriptomic landscape. Our findings may contribute to realizing future precision medicine, providing a basic rationale for the therapeutics of brain tumors.

## 1. Introduction

Brain tumors are complicated groups of neoplastic cells emerging from intracranial tissues and the meninges with grades of malignancy [1]. Brain tumors can be classified by several features like malignancy, location of origin, and genomic characteristics. A meningioma is a tumor that originated from the meninges with alterations in *NF2*, *AKT1*, *TRAF7*, *SMO*, and *PIK3C*, and most meningiomas are benign [2]. A pilocytic astrocytoma is derived from astrocytes and occurs in children with a *BRAF* alteration [2]. Ependymoma begins in the ependymal cells and occurs in young children [3]. Medulloblastoma is a usual type of brain cancer in children, and it starts from the cerebellum with alterations in *CTNNB1*, *TP53*, and *MYC* [2]. Gliomas, originating from glial progenitor cells, constitute a heterogenous group of primary central nervous system tumors, demonstrating diverse clinical presentations and pathological characteristics [4]. Lower-grade gliomas are slow growing with IDH mutation and/or 1p/19q co-deletion, and they can progress to become high-grade gliomas. Glioblastoma is the most common and lethal primary malignant brain tumor and is characterized by the absence of IDH mutations and the presence of alterations in *TERT* promoter, *EGFR*, *PDGFRA*, and *PTEN*. Brain tumors form a heterogeneous group of diseases with diverse features such as location of origin, malignancy, and age of disease onset and have intricate genomic or transcriptomic backgrounds [5]. In addition to cancer cells, the tumor microenvironment (TME) consists of immune and stromal cells, which can affect tumor progression by secreting cytokines or other inflammatory factors [6]. Although the brain has rare tissue-resident cell types including neurons, microglia, and astrocytes, the TME landscape of immune and stromal cells has been a crucial target for treating brain tumors [7,8,9]. Nevertheless, normal brain or early-stage brain tumors commonly form an immunosuppressive microenvironment, leading to numerous challenges in applying successful therapeutic methodologies. 

The standard treatment procedures for brain tumors include chemotherapy and radiotherapy in addition to surgical removal [10]. However, these approaches are not always applicable because the tumor occasionally occurs in sensitive or inoperable regions of the brain [11]. Furthermore, recurrence after treatment is common. Therefore, immunotherapy, which can alleviate some of the problems associated with the treatment of brain tumors, has evolved as an effective and safe treatment [12]. Among immunotherapeutic techniques, immune checkpoint inhibitors (ICIs) have shown good efficacy in patients with brain tumors [13]. For example, the inhibition of programmed cell death protein 1 (PD-1) or its ligand programmed death-ligand 1 (PD-L1) can affect the interaction between PD-L1 and its receptor (PD-1) to control T-cell dysfunction and to activate an immune response against tumors [13]. However, currently, the efficacy of anti-PD1/PD-L1 ICI for brain tumors is still unsatisfactory [14]. Another technique being studied to treat brain tumor patients is combined cytotoxic and immune-stimulatory gene therapy using an adenoviral vector [15]. In a recent clinical trial, combined cytotoxic and immune-stimulatory gene therapy showed tolerable toxicity and a significant increase in T cells with 21 months of median overall survival [16]. Nonetheless, another clinical trial did not lead to sufficient tumor suppression or improvement in overall survival [17]. Thus, to apply the appropriate immunotherapy and improve its effectiveness, the investigation and comparison of transcriptomic characteristics among diseases are necessary.

Recently, single-cell RNA sequencing (scRNA-seq) has advanced the field of transcriptomics by enabling high-resolution profiling of gene expression at the single-cell level [18]. scRNA-seq has provided the capability to dissect and characterize distinct cell types, transcriptional states, and dynamic cellular processes with unprecedented detail [18]. While several studies have presented insights into brain tumors using scRNA-seq, the cellular diversity in different types of tumors at the single-cell level has hardly been investigated.

In this study, we performed transcriptional profiling of different types of brain tumors, including meningioma, pilocytic astrocytoma, ependymoma, medulloblastoma, glioblastoma, and lower-grade glioma, to reveal and compare the gene expression and cellular heterogeneity across ages, malignancies, and locations of development. We estimated the TME infiltration patterns using computational algorithms. Our transcriptomic profiling will help in the research and development of therapeutics for brain tumors.

## 2. Materials and Methods

### 2.1. Collection of Datasets

Twelve microarray datasets acquired from GEO (https://www.ncbi.nlm.nih.gov/geo/ (accessed on 12 May 2022)), including GSE16581, GSE5675, GSE73066, GSE16155, GSE50385, GSE10327, GSE37418, GSE36245, GSE53733, GSE4290, GSE44971, and GSE50161, were used in this study for immune expression profiling [19,20,21,22,23,24,25,26,27,28,29,30]. Subsequently, clinical data were obtained from the Supplementary Materials or the GEOquery package (2.70.0) in R [31]. A total of 862 microarray data samples were included, and normal samples were excluded for further analysis to compare 813 tumor samples. Detailed information of microarray datasets is depicted in the Supplementary Materials (Table S1, Figure S1). Next, scRNA-seq datasets were acquired from GEO and scPortal (https://singlecell.broadinstitute.org/single_cell (accessed on 8 August 2022)), including GSE183655, GSE125969, GSE155446, GSE103224, GSE89567, and SCP271 [32,33,34,35,36,37]. The detailed information of scRNA-seq datasets is listed in Supplementary Table S2. Bulk RNA sequencing data of patients with glioblastoma who were treated with anti-PD-1 immunotherapy using pembrolizumab were also obtained from GEO (PRJNA482620, GSE121810) (Table S3) [38,39].

### 2.2. Microarray Data Processing and Immune Signature Expression Analysis 

All microarray samples used in this study were based on the Affymetrix-GPL570 platform. Background subtraction and normalization of probe set intensities were conducted using robust multi-array analysis (RMA) [40]. Background adjustment, quantile normalization, and final summarization of oligonucleotides per transcript were performed using the RMA algorithm. Probe collapsing was conducted using GSEA-P [41]. The immune signature expression was assessed using cell-type identification by estimating relative subsets of RNA transcripts x (CIBERSORTx) [42]. Data were uploaded to the CIBERSORTx web portal (https://cibersortx.stanford.edu (accessed on 3 June 2022)), and the algorithm was run using the LM22 signature and 1000 permutations. We obtained a matrix of immune infiltration patterns. The LM22 signature provided specific segregation of 22 human immune cell phenotypes, including lymphocytes and myeloid cells. The microglial signature was defined as CX3CR1, P2RY12, CSF1, CSF1R, CX3CL1, ROBO2, and CXCL14. xCell was also employed to analyze the abundance of immune signatures [43].

### 2.3. Single-Cell RNA Sequencing Data Processing

The scRNA-seq data were analyzed using the Seurat (version 4.0) R package [44]. Unique features or gene counts that exceeded 7500 or fell below 200 were excluded to eliminate the possibility of doublets or empty droplets. Subsequently, cells in which the mitochondrial genome content exceeded 30% were excluded from further analysis. The processed count matrix was log-normalized, and data integration was performed using anchors and the canonical correlation analysis (CCA) model [45]. Data scaling and linear dimensional reduction using principal component analysis (PCA) were conducted for the next steps. Cell clusters were then determined using the K-nearest neighbor (KNN) graph model and modularity optimization. Uniform manifold approximation and projection (UMAP) was used for dimension reduction and visualization. Cell-type annotation was performed according to the cell-type-specific marker genes of each cluster using the Seurat command of Findmarkers(), which detects differentially expressed genes between groups of cells using the Wilcoxon Rank Sum test.

### 2.4. Consensus Clustering Using Immune-Related Genes

We obtained immune-related genes from the immunophenogram for depicting the immune landscape of brain tumors (Table S4) [46]. Subsequently, unsupervised clustering methods (k-means) for patient stratification were used to specify immune infiltration patterns and classify samples for further analysis. The stability of the identified clusters was evaluated using a consensus clustering algorithm to determine the optimal number of clusters. The ConsensusClusterPlus (version 1.66.0) R package [47] was employed; samples were iterated 1000 times to guarantee robustness of classification. 

### 2.5. Gene Set Variation Analysis (GSVA) and Gene Set Enrichment Analysis (GSEA)

The levels of immune-related genes were quantified by gene set variation analysis (GSVA) using the R package gsva (version 1.46) [48]. The deconvolution method employed in our study entailed eight cell type classes that were divided by immunophenogram (major histocompatibility complex, immune checkpoints, activated CD4, activated CD8, T-effector memory CD4, T-effector memory CD8, myeloid-derived suppressor cell, and regulatory T cell). Next, the expression levels of mural and dendritic cell markers used in the scRNA-seq analysis were also quantified using the R package gsva. Gene set enrichment analysis (GSEA) between immune cluster 1 and immune cluster 2 was performed using the clusterprofiler (version 4.7.2) package in R [49] to evaluate the enrichment score of each gene set on the pre-ranked gene list among samples. 

### 2.6. Differentially Expressed Gene (DEG) Analysis with Gene Ontology (GO) Enrichment 

DEGs between the two clustering groups were analyzed using the R package limma (version 3.58.1) [50]. DEGs among groups were determined based on the significance criteria of *p* value < 0.05 and expression levels (|Log2 fold change| > 2). GO enrichment analysis was performed using the clusterProfiler package in R, and GO terms in biological processes were identified with an adjusted cutoff *p* value of <0.05. 

### 2.7. Visualization and Statistical Analysis 

R Studio/R4.1.2 was used for statistical analysis and data visualization. To compare two or more groups, Welch’s *t*-tests and Kruskal–Wallis tests were used to conduct unpaired statistical tests with unequal variances [51]. In addition, the independent population proportions test and Chi-square test (prop.test()), which is the R function to prove the statistical significance of differences in proportion, were used to indicate the difference in the proportion of brain tumor cell types. Box plots and bar plots were developed using the R packages ggplot2 and ggpubr, and all heatmaps were created using the pheatmap function in R.

## 3. Results

### 3.1. Landscape of the Immune Signature Expression in the TME of Brain Tumors

#### 3.1.1. Depiction of the Immune Signature Expression in Overall Brain Tumors

**Figure 1:**
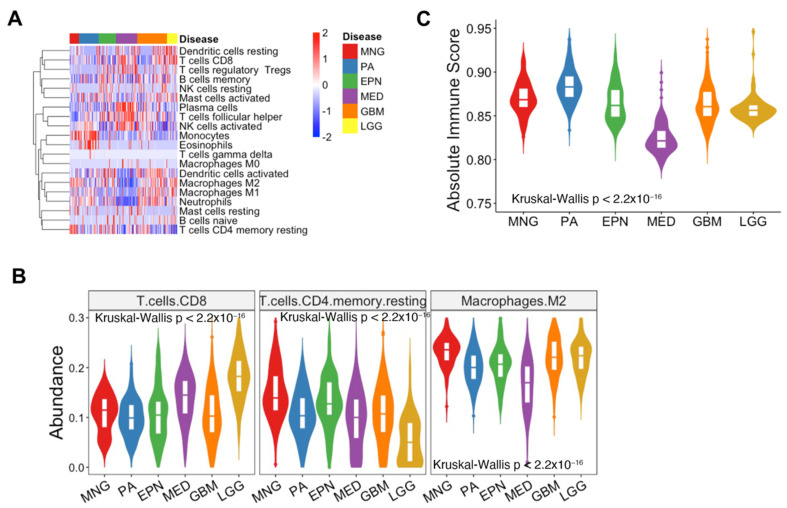
The overall immune landscape in brain tumors derived from CIBERSORTx: (**A**) Heatmap representing the overall immune signature of six types of brain tumors. (**B**) Violin plots illustrating the abundances of CD8+ T cells, CD4+ T cells, and M2 macrophages in each brain tumor type. (**C**) Violin plot showing the total immune infiltration of brain tumors from CIBERSORTx. The statistical significance was calculated using the Kruskal–Wallis test, *p* < 2.2 × 10^−16^. MNG, meningioma; PA, pilocytic astrocytoma; EPN, ependymoma; MED, medulloblastoma; GBM, glioblastoma; LGG, lower-grade glioma; CIBERSORTx, cell-type identification by estimating relative subsets of RNA transcripts x.

To analyze the TME in diverse brain tumors, we obtained microarray data of 813 samples, including meningiomas (MNGs), pilocytic astrocytomas (PAs), ependymomas (EPNs), medulloblastomas (MEDs), glioblastomas (GBMs), and lower-grade gliomas (LGGs), and employed CIBERSORTx with the leukocyte signature matrix (LM22) (Figure S2A). Among the 22 signatures, “T cell CD4 naïve” and “T cells CD4 memory activated” were excluded for further analysis as they exhibited no discernible differences between the diseases (Figure S2A). The overall immune signature of multiple cells revealed that the expression levels of B cells, T cells, natural killer cells, and myeloid cell subsets differed according to tumor types (Figure 1A). When we represented the immune signature for each brain tumor type, we found that CD8+ T cell and resting memory CD4+ T cells were more abundant than other lymphocytes, and M2 macrophages were more abundant than other myeloid cells (one-way ANOVA, *p* < 0.0001) (Figure S2B).

#### 3.1.2. Comparison of Immune Expression across Brain Tumor Types

We compared the expression of immune cells known to be associated with tumor progression as promotors or inhibitors across brain tumor types. In addition to CIBERSORTx, we also employed xCell to validate the results obtained from CIBERSORTx (Figure S2C). The abundance of M1 macrophages, which are considered to elicit an anti-inflammatory response, was higher in meningiomas, pilocytic astrocytomas, and glioblastomas (Kruskal–Wallis test, *p* < 0.0001) (Figure S2B,C) [52]. In contrast, the abundance of M2 macrophages, which help tumor growth via a pro-inflammatory response, was the highest in meningiomas and the lowest in medulloblastomas (Kruskal–Wallis test, *p* < 0.0001) (Figure 1B and Figure S2C) [52]. The abundance of CD8+ T cells was the highest in lower-grade gliomas (Kruskal–Wallis test, *p* < 0.0001) (Figure 1B and Figure S2D). Resting memory CD4+ T cells exhibited the highest abundance in meningiomas and the lowest in lower-grade gliomas (Kruskal–Wallis test, *p* < 0.0001) (Figure 1B and Figure S2C). In addition, microglia play crucial roles in brain immune responses and contribute to tumor progression by shaping the tumor microenvironment; however, the microglia signature was not included in LM22. Therefore, we defined a microglial signature and calculated the average expression levels of the microglia signature for each sample. Pilocytic astrocytomas, glioblastomas, and lower-grade gliomas showed higher expression levels of microglial signatures compared to other tumor types. In contrast, medulloblastoma exhibited the lowest expression levels. These results suggested a more frequent infiltration of microglia in astrocytic-lineage brain tumors (Figure S3). Next, the absolute total immune infiltration score illustrated that pilocytic astrocytomas showed the highest immune infiltration among the six types of brain tumor, and medulloblastomas represented the lowest immune infiltration (Kruskal–Wallis test, *p* < 0.0001) (Figure 1C). Collectively, the immune signature showed different patterns depending on the brain tumor types, and the expression of the gene signatures associated with tumor development was distinctive.

### 3.2. Single-Cell RNA Sequencing Revealed Cellular Heterogeneity in Various Brain Tumors

#### 3.2.1. The Composition of Cell Types in Brain Tumors

**Figure 2:**
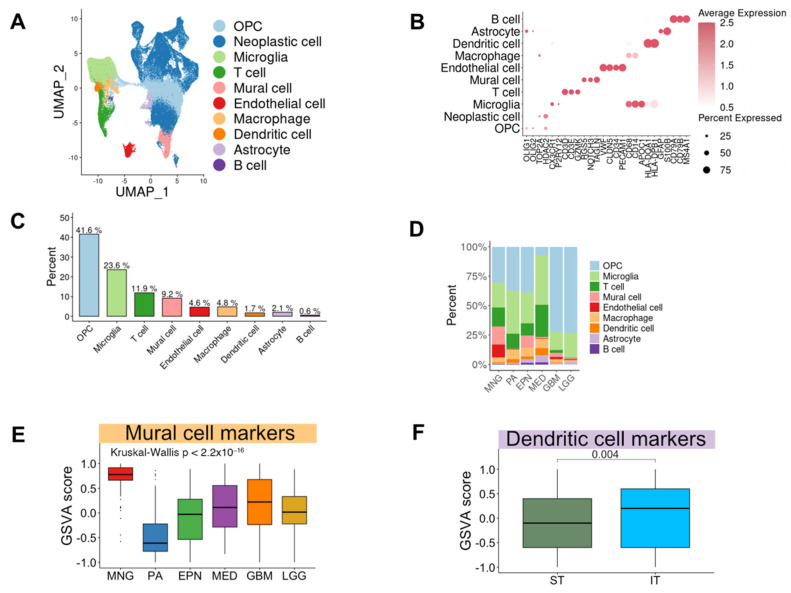
Single-cell transcriptome profiling of brain tumors: (**A**) UMAP plot of 124,967 brain tumor cells. Colors indicate cell types. (**B**) Expression of representative marker genes according to cell types. The average expression is represented by the color intensity, and the percentage of expression is shown by varying dot sizes. (**C**) Percentages of cell types in brain tumors. Colors are allocated by cell types. (**D**) Bar graph showing the proportion of cell types according to each brain tumor type. Cell types are distinguished by color, and the x-axis of the bar graph represents diseases. (**E**) GSVA score of mural cell markers in microarray data according to each brain tumor type. Brain tumor types are distinguished by color, and the y-axis shows the GSVA scores. The statistical significance was calculated by the Kruskal–Wallis test, *p* < 2.2 × 10^−16^. (**F**) GSVA score of dendritic cell markers in microarray data according to regions of origin. Locations of origin are distinguished by color, and the y-axis represents the GSVA scores. The statistical significance was calculated by a two-tailed *t*-test, *p* = 0.004. UMAP, uniform manifold approximation and projection; GSVA, gene set variation analysis; MNG, meningioma; PA, pilocytic astrocytoma; EPN, ependymoma; MED, medulloblastoma; GBM, glioblastoma; LGG, lower-grade glioma; ST, supratentorial tumor; IT, infratentorial tumor.

To depict the TME heterogeneity in brain tumors, we obtained scRNA-seq data from the GEO database and scPortal. A total of 124,967 cells passed the quality control (QC) filter, and a scatter plot obtained using UMAP revealed 24 distinct cell clusters (Figure S4A–C). The 124,967 cells were annotated into ten major cell types: oligodendrocyte progenitor cells (OPCs), neoplastic cells, microglia, T cells, mural cells, endothelial cells, macrophages, dendritic cells, astrocytes, and B cells with canonical marker genes (Figure 2A,B, Figure S4D, and Figure S5). Microglia were the largest infiltrating immune cells within the brain TME (23.6%), and T cells accounted for the second largest infiltrating immune cells (11.9%) (Figure 2C and Figure S4E).

#### 3.2.2. Difference of Cell Type Composition across Brain Tumor Types

For a precise analysis of the cell types of non-neoplastic cells in the TME across brain tumor types, we categorized cell types according to diseases. Interestingly, the cellular composition of each brain tumor type was distinctive while inter-sample variations existed (Figure 2D and Figure S4D). T cells accounted for 27% in medulloblastomas, which was higher than their levels in other brain tumor types (1638/4437 vs. 5429/48,080 cells; Chi-square test, *p* < 0.0001) (Figure 2D). The fraction of mural cells was the largest in meningiomas at 16% (3561/19,266 vs. 1904/34,835 cells; Chi-square test, *p* < 0.0001) (Figure 2D). Among immune cells, there was a higher proportion of microglia and macrophages in glioblastomas and lower-grade gliomas than other brain tumor types (3145/343 vs. 13,757/8079 cells; Chi-square test, *p* < 0.0001) (Figure S2E). A significant classification for brain tumors, which affects the surgical approach, is based on the location concerning the tentorium, which is an extension of the dura mater that divides the cerebellum from the inferior portion of the occipital lobes [53]. According to this standard, tumors were grouped based on location as supratentorial tumors (cerebrum, frontal lobe, temporal lobe, parietal lobe, and occipital lobe) and infratentorial tumors (ventricle, cerebellum, and brain stem) [54]. Accordingly, meningiomas, glioblastomas, and lower-grade gliomas were included in supratentorial tumors, and pilocytic astrocytomas, ependymomas, and medulloblastomas in infratentorial tumors. We found that the proportion of dendritic cells in infratentorial tumors was greater than that in supratentorial tumors (independent populations proportions test, *p* < 0.0001) (Figure 2D). GSVA of microarray data validated these findings (Figure 2E,F). As a result, scRNA-seq revealed an overall single-cell transcriptome atlas of brain tumors and demonstrated significant diversities of cell type composition.

### 3.3. Unique Features of Immune Subpopulations Based on Unsupervised Learning

**Figure 3:**
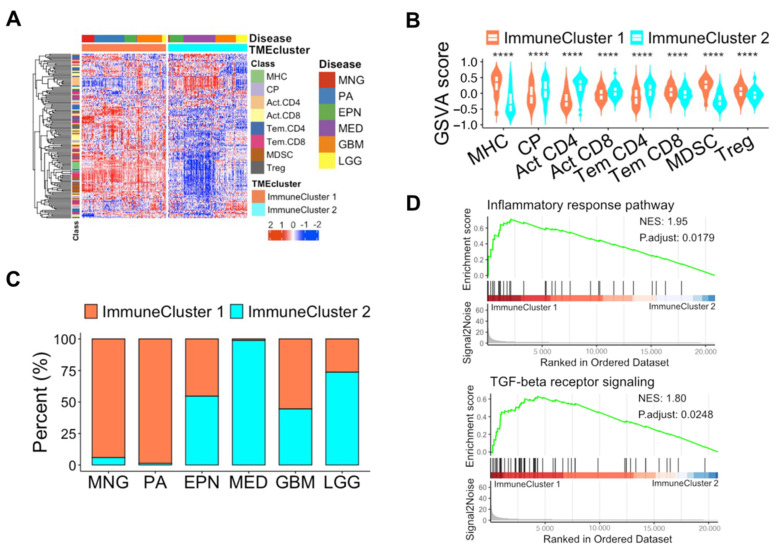
Unsupervised clustering using immune-related genes in brain tumors: (**A**) Heatmap representing the expression of immune-related genes in brain tumors. (**B**) GSVA score of classes of immune-related genes according to two distinct clusters. The statistical difference was calculated using the Kruskal–Wallis test. ****, *p* < 0.0001. MHC, major histocompatibility complex; CP, immune checkpoint; Act CD4, activated CD4; Act CD8, activated CD8; Tem CD4, T effector memory CD4; Tem CD8, T effector memory CD8; MDSC, myeloid-derived suppressor cell; Treg, regulatory T cell. (**C**) Bar plot showing the ratio of two distinct clusters by disease types. The y-axis shows the percentage of each cluster, and the x-axis shows disease types. MNG, meningioma; PA, pilocytic astrocytoma; EPN, ependymoma; MED, medulloblastoma; GBM, glioblastoma; LGG, lower-grade glioma. (**D**) GSEA plots indicating inflammatory response pathways (*p*.adjust = 0.0179, NES = 1.95) and TGF-beta receptor signaling (*p*.adjust = 0.0248, NES = 1.80) were enriched in immune cluster 1 compared to immune cluster 2. TGF, transforming growth factor.

To identify the biological and clinical characteristics, unsupervised clustering analysis using immune-related genes (Table S4) was implemented to classify patient samples into TME immune subtypes. To select the ideal cluster number, we examined the clustering stability using the ConsensusClusterPlus R package (Figure S6) [47]. The unsupervised clustering analysis separated the total samples into two distinct clusters: immune cluster 1 and immune cluster 2 (Figure 3A). Immune cluster 1, characterized by higher total absolute immune scores, had higher GSVA scores of major histocompatibility complex (MHC), T effector memory CD8 (Tem CD8), myeloid-derived suppressor cell (MDSC), and regulatory T cells (Tregs), and immune cluster 2 had higher GSVA scores of immune checkpoints (CP), activated CD4 (Act CD4), activated CD8 (Act CD8), and T effector memory CD4 (Tem CD4) (Figure 3B and Figure S7A). Immune cluster 1 accounted for most of the meningiomas and pilocytic astrocytomas, and immune cluster 2 accounted for most of the medulloblastomas (Figure 3C). Other tumor types were almost equally distributed into immune clusters 1 and 2 (Figure 3C). 

To better understand the biological characteristics, we performed GSEA. We found that the inflammatory response pathway and transforming growth factor (TGF) beta receptor signaling pathway were enriched in immune cluster 1 (Figure 3D). These results suggested that unsupervised clustering using immune-related genes was a promising biomarker for patient stratification with distinctive immune-related characteristics.

Furthermore, to assess the clinical relevance of the immune clusters, we performed Kaplan–Meier analysis using progression-free survival (PFS) data from glioblastoma (GSE36245) and ependymoma (GSE16155) datasets, for which clinical information was available. The Kaplan–Meier analysis of PFS revealed that immune cluster 1 showed significantly longer PFS than immune cluster 2 for patients in both the GBM and EPN datasets who received standard treatment (Figure S7B). This result might be attributed to the overall dominance of immune infiltration in immune cluster 1, characterized by a high expression of MHC and T effector memory CD8.

### 3.4. Immune Subpopulations were Potential Biomarkers of Reaction to PD-1 Blockade in Glioblastomas

**Figure 4:**
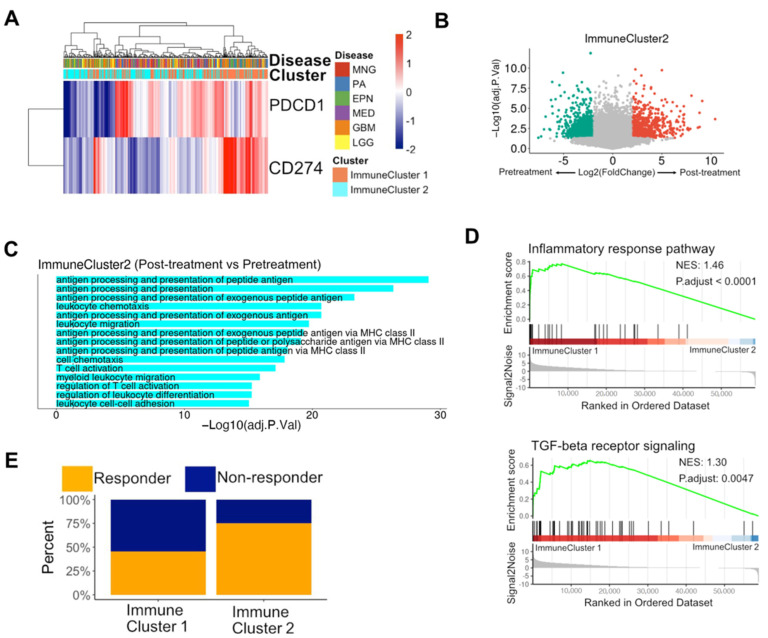
Reaction after PD-1 blockade depends on immune-related unsupervised clustering in glioblastoma: (**A**) Heatmap representing PD-1 and CD274 (PD-L1) expression among samples of brain tumors. (**B**) Volcano plot showing differentially expressed genes (DEGs) between post-treatment and pre-treatment samples in immune cluster 2. Color dots represent DEGs (|Log2FoldChange| > 2, adjusted *p*-value < 0.05). (**C**) Gene ontology (GO) enrichment in DEGs between post- and pre-treatment samples. The x-axis indicates the –log10-adjusted *p*-value. (**D**) GSEA plots denoting the inflammatory response pathway (*p*.adjust < 0.0001, NES = 1.46) and TGF-beta receptor signaling (*p*.adjust = 0.0047, NES = 1.30) were enriched in immune cluster 1 compared to immune cluster 2. (**E**) Percentages of response rate after anti-PD1 treatment in glioblastoma patients stratified by unsupervised clustering. TGF, transforming growth factor; GSEA, gene set enrichment analysis; NES, normalized enrichment score.

We examined *PDCD1* (PD-1) and *CD274* (PD-L1) expression in patient samples and found that the expression levels of *PD-1* and *CD274* were distinguished according to immune subpopulations (Figure 4A). To understand if immune subpopulations could be used as latent biomarkers of reaction to anti-PD-1 treatment, we analyzed two publicly available bulk RNA-seq datasets that included data of patients with glioblastomas who received anti-PD-1 treatment using pembrolizumab [38,39]. For patient classification, we separated the samples into two distinct clusters using unsupervised clustering with immune-related genes. By performing DEG and GO analyses between post-treatment samples and pre-treatment samples, we found that the DEGs up-regulated in post-treatment samples were associated with GO terms of antigen processing and presentation with T-cell activation in immune cluster 2 but not in immune cluster 1 (Figure 4B,C and Figure S8A,B). In addition, immune cluster 2 elicited a better clinical response rate than immune cluster 1 (45% vs. 75%) (Figure 4E). The discrepancy in clinical responses between standard therapy and anti-PD1 treatment in GBM implied that patients in immune cluster 2 might not obtain clinical benefits from standard therapy but could benefit from immunotherapy including anti-PD1, with elevated antigen processing and presentation with T-cell activation under treatment. GSEA results of the two datasets indicated that inflammatory response and TGF-beta-receptor signaling-related pathways were significantly enriched in immune cluster 1 compared to immune cluster 2 (Figure 4D and Figure S8C). Altogether, these results suggested that immune subpopulations could be potential predictive biomarkers of response to anti-PD-1 treatment for patients with glioblastoma.

## 4. Discussion

Brain tumors are associated with only a small proportion of annual cancer incidence (1.4%), but these tumors account for almost double the proportion (2.7%) of tumor-related deaths [12]. To overcome the limitations of brain tumor therapies, several clinical studies have focused on the key players in the brain TME [12]. Here, we investigated the complex transcriptomic structures in multiple brain tumor types to highlight latent strategies to treat brain tumor patients and surmount therapeutic limitations.

Our analyses unveiled that T-cell and macrophage signatures were highly expressed in the overall brain TME, especially CD8+ T cells, CD4+ T cells, and M2 macrophages. These three cell types are significant contributors to tumor biology. CD8+ T cells release interferon-*γ* to elicit an anti-tumor immune response, and CD4+ T cells form a part of the adaptive immune system and are expressed on the host cell surface bound to MHC molecules [55]. Additionally, M2 macrophages form an immunosuppressive microenvironment and play a pro-tumorigenic role, secreting cytokines like interleukin-4 (*IL-4*), interleukin 10 (*IL-10*), and interleukin 13 (*IL-13*) [52]. Thus, reinforcing T cells or suppressing M2 macrophages could assist in the treatment of patients with brain tumors. Moreover, scRNA-seq revealed that T cells were the most abundant in medulloblastomas, and macrophages and microglia were more enriched in glioblastomas and lower-grade gliomas among immune cells. Consistently, in a previous study, T cells were more enriched in medulloblastomas than pilocytic astrocytomas and glioblastomas [56]. Another previous study disclosed that tumor-associated macrophages and microglia (TAMs) emerge as the predominant population of immune cells within the glioblastoma microenvironment [57]. Previous studies have shown that brain-resident microglia and peripheral macrophages are known to infiltrate brain tumors and present immunosuppressive signals [12]. Our integrative analysis strengthens the information on cell type compositions in diverse brain tumors and suggests that T-cell manipulation could improve the efficacy of immunotherapy for medulloblastomas, and the regulation of macrophages and microglia would be an effective strategy to treat glioblastomas and lower-grade gliomas. 

scRNA-seq decomposed the cellular heterogeneity of immune cells as well as other significant cellular populations. Here, we provide a large single-cell transcriptome atlas of brain tumors and illustrate the significant distinctions in cellular composition among brain tumor types. The proportion of mural cells in meningiomas was higher than that in other diseases. This could be because it has been previously reported that the brain cortex contains more mural cells than other regions, including the cerebellum, midbrain, and hippocampus [58]. Additionally, a previous single-cell transcriptome profiling study of meningiomas revealed the presence of mural cells [59]. Mural cells are notable regulators of tumor vessel formation and are considered modulators of tumor angiogenesis and growth [60]. Hence, mural cells that line the tumor blood vessels are critical targets of angiogenic inhibitors. This result suggests that anti-angiogenesis therapy in meningiomas may lead to better efficacy than in other brain tumor types. Another major finding is that the abundance of dendritic cells was higher in infratentorial tumors than in supratentorial tumors. Dendritic cells exist within the brain, where they behave as observers during brain disease and neuroinflammation [61]. In the TME, dendritic cells present tumor-associated antigens on MHC molecules to encourage T-cell responses [62]. Therefore, vaccination with dendritic cells laden with immunogenic cell-death-driven tumor antigens could help improve the efficacy of PD-1 treatment [62]. This result implies that exploiting dendritic cells in immunotherapy could lead to better treatment efficacy for infratentorial tumors. 

The detailed TME signature Is a biomarker for the prognoses of patients with brain tumor and for developing a more impactful therapeutic approach. To understand the TME landscape in brain tumors or other regions, previous studies carried out unsupervised clustering analysis with immune-related features for patient stratification and recapitulated the heterogenous characteristics [63,64,65]. In our study, unsupervised clustering based on immune-related genes showed that brain tumor patients could be separated into two distinct subpopulations with distinctive transcriptomic features. After we employed the same algorithm on patients with glioblastomas after anti-PD1 treatment using pembrolizumab, which is a humanized monoclonal immunoglobulin G4 (IgG4) anti-PD1 antibody, we found that the functions of DEGs between post-treatment and pre-treatment, the enrichment of pathways related to tumor progression, and clinical responses to anti-PD1 treatment were considerably different, suggesting that immune-related patient stratification could be a latent biomarker of reaction and clinical response to the PD-1 blockade in glioblastomas. This result might be affected by inflammatory responses and TGF-beta signaling in immune cluster 1 caused by MDSCs and Tregs and higher GSVA scores of activated T cells with elevated antigen processing and presentation with T-cell activation under treatment in immune cluster 2. Furthermore, previous studies have shown that pilocytic astrocytomas exhibit a significantly lower percentage of CD8+ T cells and NK T cells than glioblastomas, and PD-1+/CD4+ T cells are more enriched in medulloblastomas than in pilocytic astrocytomas [30,66]. Given that pilocytic astrocytomas were enriched in immune cluster 1 and medulloblastomas were enriched in immune cluster 2, the cellular composition of the TME strongly affects immunotherapy response. In particular, medulloblastomas, distinguished by high T-cell infiltration and immune cluster 2 enrichment, could be anticipated to exhibit a more favorable response to immunotherapy than other types of brain tumors.

Despite our efforts to discover distinctive characteristics of diverse brain tumor types by comprehensive transcriptomic profiling, there are some limitations that should be addressed. Our study is a retrospective analysis conducted by integrating publicly available transcriptomic data. Consequently, the analysis has limitations including sample bias and a lack of clinical implications. Nonetheless, our study suggests latent therapeutic strategies to cure brain tumors by recapitulating the brain tumor microenvironment and has the potential to lead to more comprehensive and targeted research.

In summary, we conducted an overall transcriptome profiling of multiple types of brain tumors and provided a distinct TME landscape across brain tumor types. We present potential evidence to improve treatment methods for patients with brain tumors. 

## 5. Conclusions

Our study revealed that T-cell and macrophage signatures were highly expressed in the brain TME and T cells were significantly enriched in medulloblastomas. In addition, macrophages and microglia were predominant populations in glioblastomas and lower-grade gliomas. scRNA-seq analysis showed that the proportion of mural cells in meningiomas was higher than that in other diseases and the abundance of dendritic cells was higher in infratentorial tumors. Lastly, unsupervised clustering with immune-related genes represented two distinct subpopulations with transcriptomic and clinical features. In conclusion, comprehensive transcriptomic profiling of brain tumors showed the dynamic characteristics of immune cells as well as stromal cells and distinctive molecular features of immune subpopulations, which may help improve therapeutic strategies for patients with brain tumors. 

## Data Availability

A publicly available database was used. All codes used for this study are described in GitHub (jiin04018/BT_profiling).

## Supplementary Materials

The following supporting information can be downloaded at https://www.mdpi.com/article/10.3390/biomedicines12030506/s1: Figure S1: Sample information of microarray datasets; Figure S2: Immune signature abundance of brain tumors; Figure S3: Signature expression of microglia in brain tumors; Figure S4: Distribution of cells in brain tumors; Figure S5: Expression of cell type markers; Figure S6: Consensus cluster matrices of K; Figure S7: Characteristics of immune clusters; Figure S8: Results of analysis of differentially expressed genes and gene set enrichment analysis after anti-PD1 treatment in patients with glioblastoma; Table S1: Information of microarray data; Table S2: Information of single-cell RNA sequencing data; Table S3: Information of bulk RNA-seq data; Table S4: List of immune-related genes.
