# Supplementary material for: Comprehensive Transcriptomic Profiling of Diverse Brain Tumor Types Uncovers Complex Structures of the Brain Tumor Microenvironment
Source: Biomedicines. 2024 Feb 23;12(3):506. doi: 10.3390/biomedicines12030506 (PMC10967799; doi:10.3390/biomedicines12030506)
Supplement: Supplementary file 1 [file biomedicines-12-00506-s001.zip › biomedicines-2808991-supplementary.pdf]

Supplementary Materials

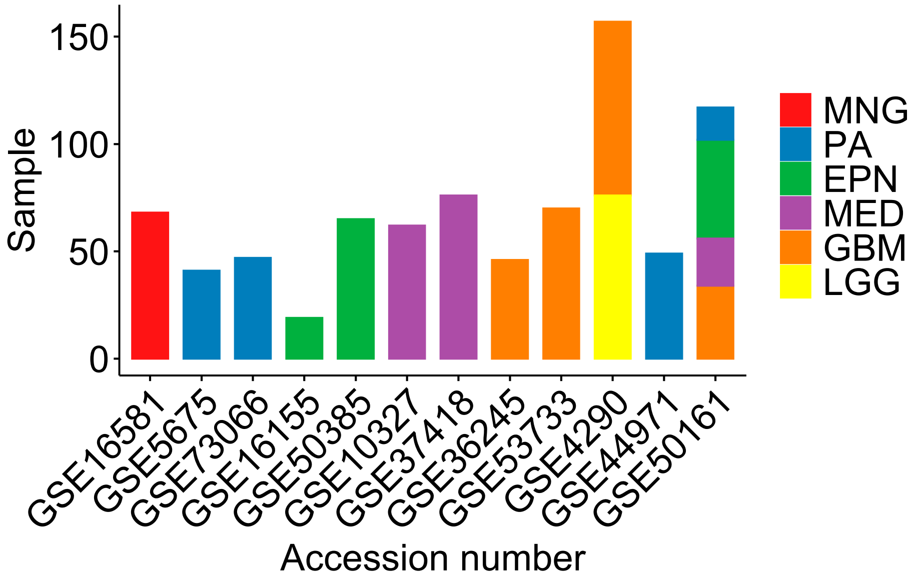

Figure S1. Sample information of microarray datasets.

A bar plot representing the size and composition of each dataset. The X-axis shows the accession number of each dataset, and Y-axis shows the number of samples.

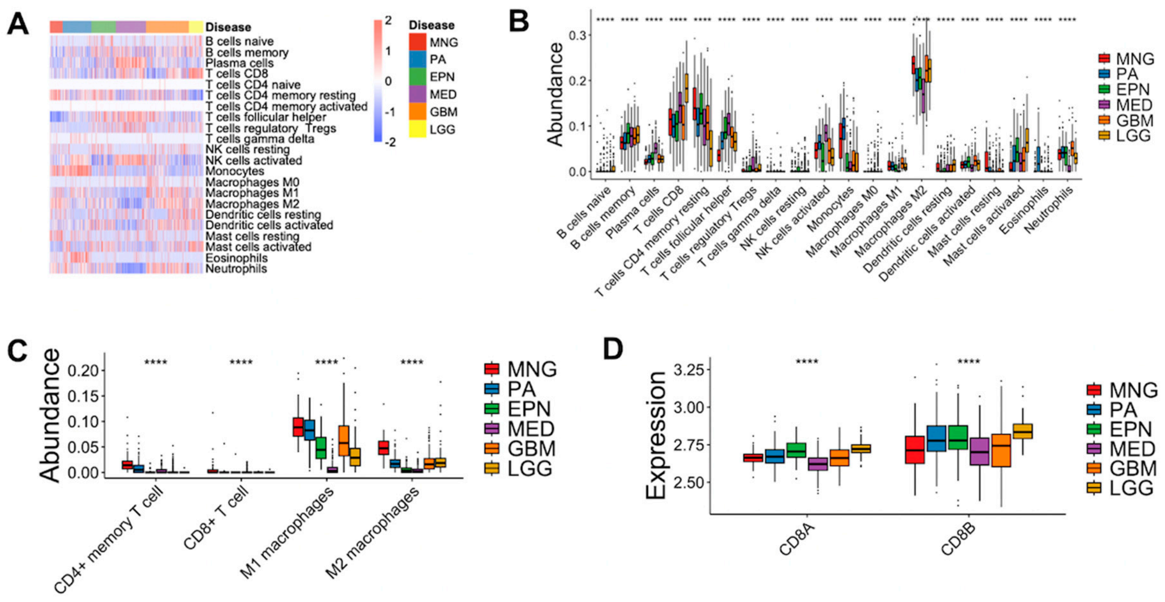

Figure S2. Immune signature abundance of brain tumors.

(A) Heatmap representing the LM22 signatures of six types of brain tumors obtained from CIBERSORTx.

(B and C) Boxplot illustrating the immune signature expression abundance of each brain tumor type by CIBERSORTx (B) and xCell (C).

(D) Box plots showing the expressions of *CD8A* and *CD8B*. ****, $P < 0.0001$.

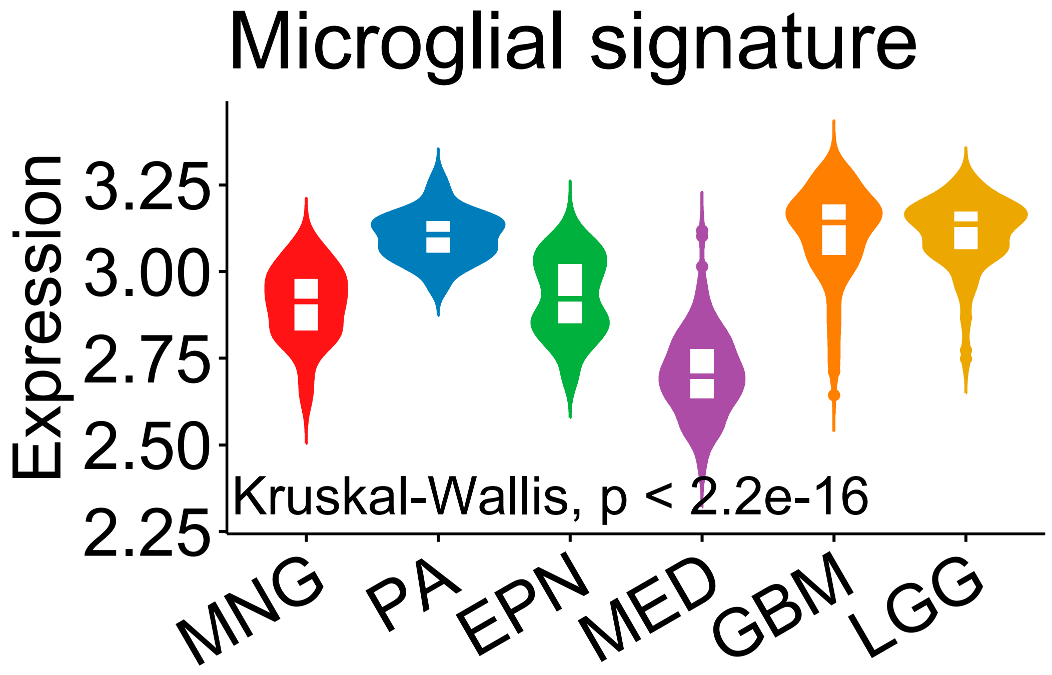

Figure S3. Signature expression of microglia in brain tumors.

Violin plot representing the average expression of signature genes of microglia (CX3CR1, P2RY12, CSF1, CSF1R, CX3CL1, ROBO2, CXCL14).

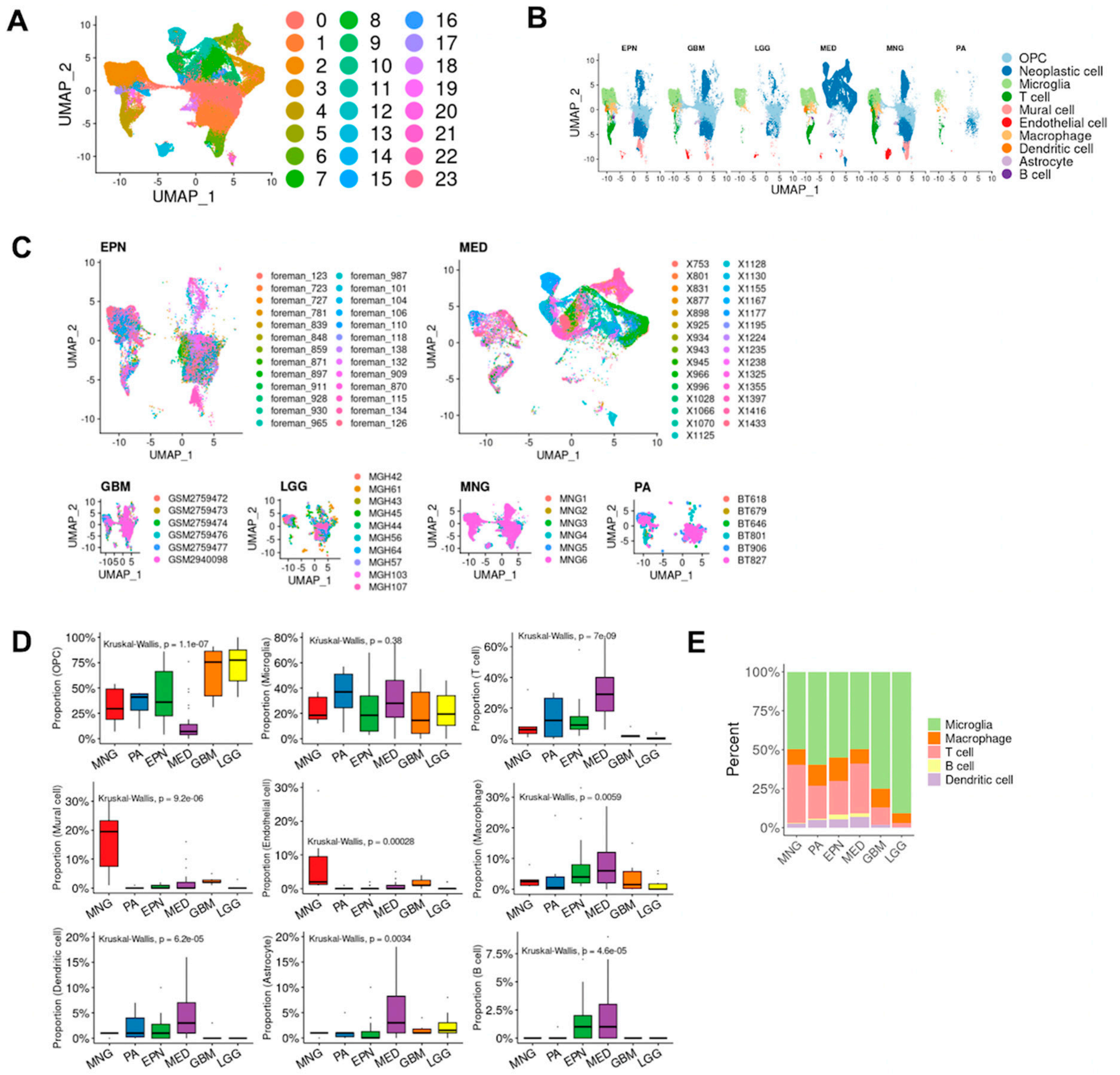

Figure S4. Distribution of cells in brain tumors.

(A) UMAP plot of 124,967 cells of brain tumor. Colors indicated distinctive clusters.

(B) UMAP plot of scRNA-seq data divided into each brain tumor type. Colors indicated cell types.

(C) UMAP plot of scRNA-seq data divided into each sample. Colors indicated samples.

(D) Boxplots illustrating the cell type proportions in each disease. Colors indicated diseases.

(E) Bar graph representing the proportion of immune cell types according to each brain tumor type. Cell types were distinguished by color.

UMAP, Uniform Manifold Approximation and Projection; scRNA-seq, single-cell RNA sequencing

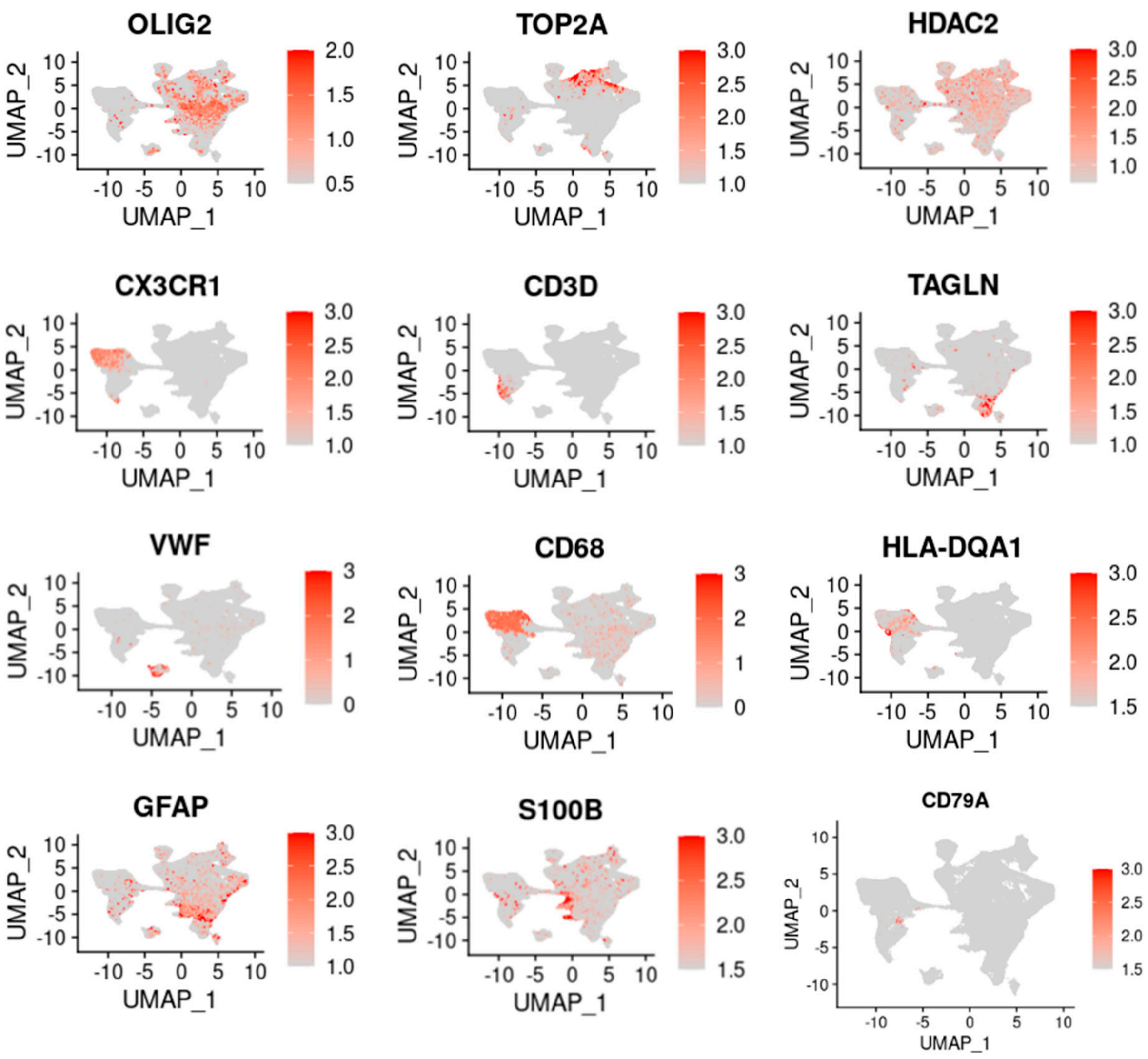

Figure S5. Expression of cell type markers.

UMAP plot representing the cell type marker gene expressions.

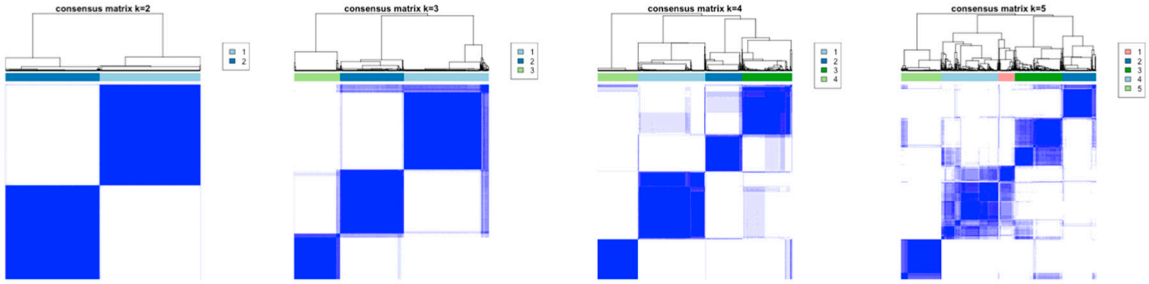

Figure S6. Consensus cluster matrices of K.

Consensus matrixes of all brain tumor samples for each K (k = 2-5), showing the clustering stability using 1000 iterations of hierarchical clustering.

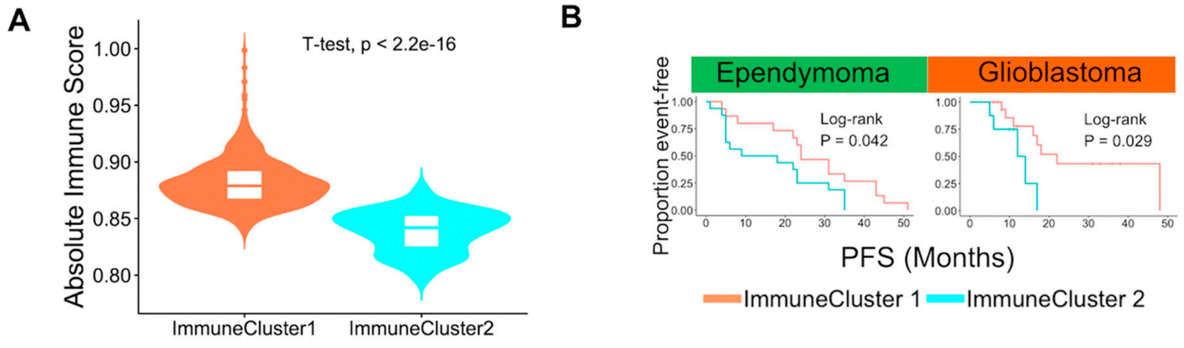

Figure S7. Characteristics of immune clusters.

(A) Violin plot representing the absolute immune scores of immune subtyping models calculated by CIBERSORTx.

(B) Kaplan-Meier plot showing the Progression-free survival of patients with GBM and EPN between immune cluster 1 and immune cluster 2.

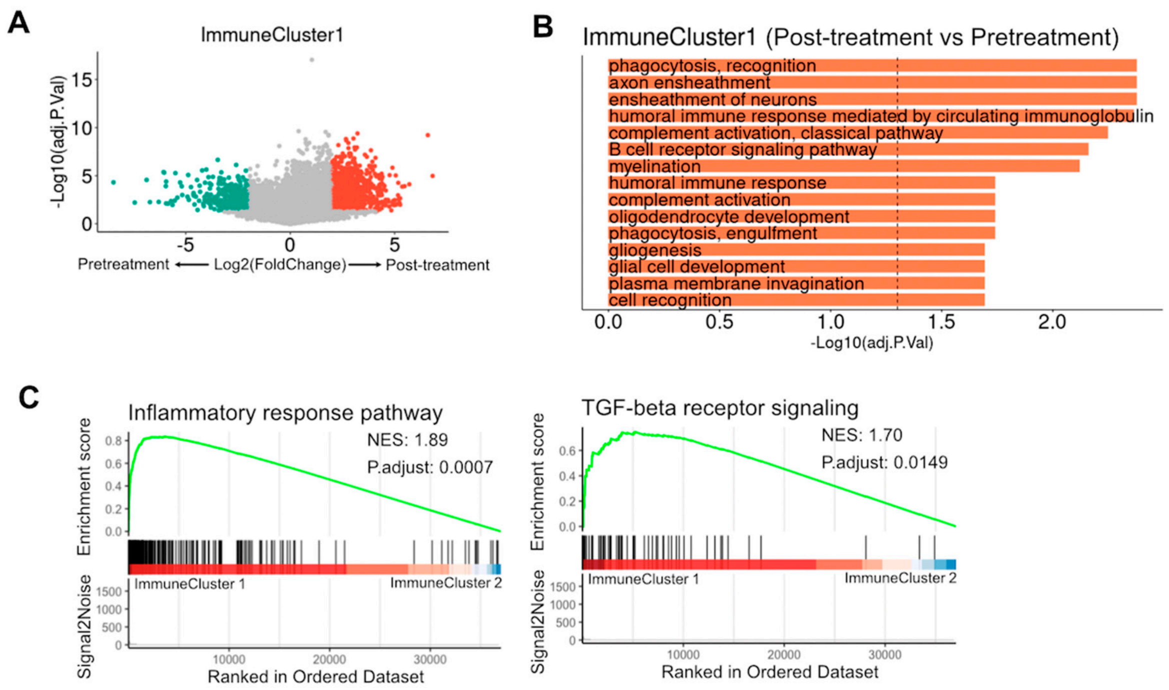

Figure S8. Results of analysis of differentially expressed genes and gene set enrichment analysis after anti-PD1 treatment in patients with glioblastomas.

(A) Volcano plot showing differentially expressed genes (DEGs) of post-treatment samples and pretreatment samples in immune cluster 1 (|Log2FoldChange| > 2, adjusted P-value < 0.05).

(B) Gene ontology (GO) enrichment in DEGs post-treatment samples versus pretreatment samples in immune cluster 1. X-axis indicated –log10 adjusted P-value.

(C) GSEA plots denoting inflammatory response pathway (P.adjust = 0.0007, NES = 1.89), TGF-beta receptor signaling (P.adjust = 0.0149, NES = 1.70) were enriched in immune cluster 1 compared to immune cluster 2.
